# Study protocol for a single-centre observational study of household wellbeing and poverty status following a diagnosis of advanced cancer in Blantyre, Malawi - *‘Safeguarding the Family’ *study

**DOI:** 10.12688/wellcomeopenres.15633.2

**Published:** 2020-03-02

**Authors:** Maya Jane Bates, Adamson Muula, Stephen B. Gordon, Marc Y.R. Henrion, Ewan Tomeny, Peter MacPherson, Bertel Squire, Louis Niessen

**Affiliations:** 1 University of Malawi College of Medicine, P/Bag 360, Blantyre 3, Malawi; 2 Liverpool School of Tropical Medicine, Pembroke Place, Liverpool, L3 5QA, UK; 3 Malawi Liverpool Wellcome Trust, P O Box 30096, Blantyre 3, Malawi; 4 London School of Hygiene and Tropical Medicine, Keppel Street, London, WC1E 7HT, UK

**Keywords:** Out of pocket, cost of illness, economic burden, cancer, palliative, Malawi, Africa, non-communicable disease

## Abstract

**Background**: Many households in low-and-middle income countries face the additional burden of crippling out-of-pocket expenditure when faced with a diagnosis of life-limiting illness. Available evidence suggests that receipt of palliative care supports cost-savings for cancer-affected households. This study will explore the relationship between receipt of palliative care, total household out-of-pocket expenditure on health and wellbeing following a first-time diagnosis of advanced cancer at Queen Elizabeth Central Hospital in Blantyre, Malawi.

**Protocol**: Patients and their primary family caregivers will be recruited at the time of cancer diagnosis.  Data on healthcare utilisation, related costs, coping strategies and wellbeing will be gathered using new and existing questionnaires (the Patient-and-Carer Cancer Cost Survey, EQ-5D-3L and the Integrated Palliative Care Outcome Score). Surveys will be repeated at one, three and six months after diagnosis. In the event of the patient’s death, a brief five-item questionnaire on funeral costs will be administered to caregivers not less than two weeks following the date of death. Descriptive and Poisson regression analyses will assess the relationship between exposure to palliative care and total household expenditure from baseline to six months. A sample size of 138 households has been calculated in order to detect a medium effect (as determined by Cohen’s f ^2^=0.15) of receipt of palliative care in a regression model for change in total household out-of-pocket expenditure as a proportion of annual household income.

**Ethics and dissemination**: The study has received ethical approval. Results will be reported using STROBE guidelines and disseminated through scientific meetings, open access publications and a national stakeholder meeting.

**Conclusions**: This study will provide data on expenditure for healthcare by households affected by advanced cancer in Malawi. We also explore whether receipt of palliative care is associated with a reduction in out-of-pocket expenditure at household level.

## Introduction

The impact of a diagnosis of cancer on households in low and middle-income countries (LMICs) is dramatic. A study of over 9000 cancer patients in South East Asia reported that 75% of patients had either died or faced financial catastrophe twelve months from diagnosis ^1^. In African settings, cancer is associated with high mortality, as well as catastrophic financial, psychological and spiritual morbidity ^2, 3^. Many households experience cancer diagnoses when they would expect to be at their most economically productive ^4^. For the few patients who are able to access potentially curative cancer therapy, treatment abandonment rates are high ^5, 6^.

The Lancet Commission on Palliative Care and Pain Relief states that ‘ *access to palliative care and pain relief is a health, equity and human rights imperative which has been largely ignored in the goal to achieve Universal Health Coverage (UHC)’ ^7^.* Palliative care is an approach which improves quality of life of patients and families affected by life limiting illnesses ^8^. Provision of palliative care should not be limited to those thought to be in ‘terminal’ or ‘end of life’ situations; these terms lack clear definition and risk a ‘missed opportunity to do better for patients’ ^9^. Cost savings have been associated with a variety of models of delivery of palliative care, though the majority of data are reported from high income settings, and from a health systems rather than patient perspective ^10, 11^.

Out-of-pocket expenditure (OOPE) accounts for 23% of global health expenditure and 45% of health expenditure in the developing world ^12^. In Malawi – where the Essential Health Package (EHP) is provided at no cost to users at the point of care – OOPE remains a significant burden on rural households, accounting for an estimated 13–22% of health expenditure ^13^. Interventions aimed at reducing the burden of non-communicable diseases can play a key role in global development, facilitating progress towards the Sustainable Development Goals including – and beyond – ensuring healthy lives ^14– 16^.

In this study we will explore the association between receipt of palliative care and total household expenditure on health (as a proportion of total household income), and wellbeing for those who were being diagnosed with cancer for the first time, and who were assessed clinically to have advanced Kaposi’s sarcoma, cervical or oesophageal cancer or hepatocellular carcinoma at Queen Elizabeth Central Hospital in Blantyre, Malawi. We hypothesised that, as a result of pain and symptom management and provision of information about their condition, patients receiving palliative care will maintain or improve their wellbeing whilst requiring fewer repeat visits to health providers. In this way, receipt of palliative care will be associated with a reduction in total household expenditure on health over time, whilst patient (and carer) wellbeing is maintained or improved.

## Protocol

### Details of ethical issues and ethical approval received

This study has undergone ethical review by, and received approval from, the College of Medicine Research Ethics Committee in Blantyre, Malawi (P.05/18/2395) and the Liverpool School of Tropical Medicine Research Ethics Committee (18/046). All participants (patients and household carers) will be invited to give written informed consent to take part in the study. All electronic and paper-based data will be anonymised.

### Setting

Malawi is a low-income country in Central Southern Africa. Health services are provided free at the point of care through a network of community and hospital-based services supported by government and faith-based funding across 28 districts ^17^. There are four publicly funded tertiary referral (‘central’) hospitals situated in the cites of Zomba, Lilongwe, Blantyre and Mzuzu. Queen Elizabeth Central Hospital (QECH) in Blantyre is the largest central hospital in the country offering specialist services for gynaecological oncology, oncology, endoscopy and palliative care. Oncology services are at an early stage of development with limited specialist capacity and no radiotherapy available in-country ^18^. Tertiary level palliative care services ^19^ have been established for adults and children for over fifteen years, and are delivered through in-patient referral, out-patient clinics, and community based care ^20, 21^. Recruitment for this study will take place amongst adults (anyone 18 years and above) from in-patient wards and out-patient clinics (oncology, endoscopy, gynaecological oncology and palliative care) at QECH. Patients with hepatocellular carcinoma will be identified through enhanced case finding via an ongoing study on hepatitis B taking place at the same institution.

### Participant identification, recruitment and follow-up

Patients with a first-time clinical diagnosis of advanced Kaposi’s Sarcoma (KS), or cervical or oesophageal cancer will be approached at the site of specialist clinical service (oncology, gynaecological oncology, palliative care) at Queen Elizabeth Central Hospital (QECH). Patients with hepatocellular carcinoma (HCC) will be approached via referral from the study team. Eligibility screening of all patients will be undertaken by the Principal Investigator, following which patients will be provided with a study information sheet and invited to provide written consent. Eligibility criteria, information sheets and consent forms have been provided as *Extended data* ^22^. Once a patient has given consent, they will be invited to identify up to four household carers who may be approached to take part in the study. Any (or all) of these carers will be approached as soon as possible after patient recruitment, screened for eligibility, and provided with information before being invited to provide written consent. Eligibility screening, consent and baseline data collection will take place at the hospital, with subsequent data collection taking place either at hospital or at the preferred place of the participants, either home, hospital or local health centre. 

In the event of patient death, a household member (either the carer already consented, or an alternative person identified by the previously consented carer) will be invited to consent to complete a brief five-item questionnaire on funeral costs. This data will be gathered no less than two weeks following the death of the patient.

### Inclusion: cancer types and diagnostic criteria

Three cancer types (KS, cervical, oesophageal) have been selected because they have the highest incidence in the local setting ^23, 24^, and because they are amenable to clinical diagnosis under routine care at specialist clinics at QECH. Hepatocellular carcinoma is another common malignancy ^25^, which is currently under surveillance on the medical wards at QECH as part of an ongoing study on hepatitis B in Malawi. Recruitment will rely on clinical diagnosis under the supervision of specialist clinicians, as standard of care for diagnosis in the local setting ( Table 1) for criteria used for diagnosis of ‘advanced’ cancer, according to disease type). Histological confirmation is not mandatory, waiting for biopsy result would result in significant delays ^26^. Cancer staging will be recorded where available.

**Table 1.** Diagnostic criteria for advanced cancer.

Cancer type	Diagnostic criteria for advanced disease
Kaposi’s sarcoma	ALL patients with a first-time diagnosis on clinical examination by specialist doctor AND assessed as AIDS Clinical Trials Group (ACTG) ‘poor risk’ category OR ALL with a first-time diagnosis of KS where staging not done
Cervical cancer	ALL patients with a first-time diagnosis on clinical examination by specialist doctor AND with disease at International Federation of Gynaecology and Obstetrics (FIGO) stage 2 and above
Oesophageal cancer	ALL patients with a first-time diagnosis on endoscopy by specialist doctor AND assessed as being inoperable,
Hepatocellular carcinoma	ALL patients identified with a liver mass followed by confirmatory ultrasound with mass >2cm performed by a specialist doctor AND evidence of local mass effect.

### Study tools

Prior to this study, preliminary work was undertaken to explore household concepts of wellbeing and cost areas of importance to patients following a diagnosis of advanced cancer ^27^. Following this, we adapted the WHO TB patient cost survey for a cancer population as the Patient-and-Carer Cancer Cost (PaCCCt) survey, details of this process and resulting survey content have been reported elsewhere ^28^. A locally validated Chichewa language translation of the EQ-5D-3L (paper based) and the Integrated Palliative Care Outcome Scale (IPOS http://pos-pal.org, tablet based) will be used to record changes in wellbeing over time. All newly developed content in the surveys have been translated into Chichewa and piloted amongst patients and carers receiving palliative care for advanced cancer ^28^. Multiple reviews of these questions were conducted with experienced fieldworkers during transition from paper-based to tablet-based format. New questions have also been back translated for quality control by the Malawi-Liverpool-Wellcome Trust Clinical Research Programme (MLW) Translation Unit to ensure consistency of questioning during data collection.

The PaCCCt survey records details of healthcare utilisation firstly from time of onset of symptoms (reported at the time of diagnosis i.e. baseline visit) and subsequently from the last study visit (for follow up visits). Households are asked to provide details of frequency of visits, type of provider, and length of visit (including transportation to and from provider) as details necessary for calculation of all direct and indirect household expenditure on health. Visits to conventional (hospital and health centre) and non-conventional (traditional healers, drugstores) healthcare providers will be recorded. At subsequent follow-up study visits, details of emergency (unplanned) and routine (planned) visits will be recorded separately. Coping strategies (including loans and dissaving) and sources of funding for healthcare utilisation are also recorded. Household income will be captured by self-report and via use of an asset score based on a locally developed proxy means test for poverty. The Malawi Urban Proxy Means Test for Poverty was originally developed by Payongayong *et al.* ^29^ The parameters for this test have recently been updated by one of us (PM) using data from the 2016–2017 Integrated Household Survey.

### Primary outcome

Change in total household OOPE on health, as a proportion of annual household income (based on income before the onset of illness) from diagnosis to six months.

### Secondary outcomes

Change in index health status and/or frequencies by level within dimensions within the EQ-5D-3L and/or visual analogue scores from time of diagnosis to six months.

Changes in symptom burden and/or self-reported experience of physical/psychological/spiritual symptom burden using Integrated Palliative Care Outcome Scale.

Study results will be reported using the STROBE guidelines.

Receipt of palliative care will be presented as both categorical (yes [any] / no [none]) variables, or as interval data (based on the number of contacts with palliative care services from baseline to six months). Proportion of households likely to receive a referral to palliative care services will be dependent on the routine practice of the assessing clinician. 

Poverty status will be derived from two approaches, firstly self-reported household income before the onset of symptoms and secondly from a proxy means test for poverty (derived from Malawi Demographic Health Survey 2006–7) based on household assets. Household poverty status will be presented as tertiles – least poor, poor and non-poor.

Mean (and confidence intervals) and median (and inter-quartile ranges) values will be used to describe the characteristics of the study cohort for continuous variables. Categorical variables will be summarised by frequency tables and bar charts. 

The effect of receipt of palliative care on household expenditure will be estimated using a multiple linear regression model, adjusted for expenditure at diagnosis and other potential confounding variables (age, sex, rural/urban dwelling).

Poisson regression will be used to assess the relationship between the number of palliative care visits and change in total household expenditure on health (as a proportion of total household income) from the time of diagnosis to six months.

The unadjusted hazard of death will be estimated using the Kaplan-Meier survival estimator. Survival will be disaggregated by poverty status, sex, number of palliative care visits and cancer type. Cox proportional hazard models will investigate risk factors for death by calculating hazard ratios and 95% confidence intervals. The validity of the proportional hazards assumption for the Cox models will be tested. Log rank tests will be used to test for difference in survival curves between different groups.

### Sample size

The sample size calculation for this study was powered to detect a medium effect (as determined by Cohen’s f ^2^=0.15) of a predictor in a multiple linear regression model for change in total household OOPE as a proportion of annual household income. All power analyses are based on alpha = 0.05; power=0.8, using two sided tests.

Comparing single variables (e.g. response variable = change in total household OOPE as a proportion of annual household income, predictor = receipt of palliative care) a sample size of 55 households is required to detect a medium effect. Considering 50% exclusions and 20% dropout a sample size of 138 households is required (n= 55 / ((1-0.5)*(1-0.2)) = 138).

Following review of local data from relevant clinics at QECH, an estimated 225 patients are available for recruitment over a six-month period.

### Loss to follow-up

Loss to follow-up may be high in this study population, who have a diagnosis of advanced cancer with a high risk of mortality over the six-month study period. At the time of recruitment participants will be asked to provide details of directions to reach their household and asked their preferred place for follow-up visits. If they are absent from home on the first home visit, a further two visits will be attempted by the research team before declaring the household lost to follow-up. Phone contact will be tried a maximum of three times before a participant household will be considered lost to follow-up.

Participant households will be given 500MK ($0.75) mobile airtime at each visit to use in the event of any change of address or patient status during the study. In addition, the study team will call patients and their carers between scheduled visits to check on status and current place of residence. In accordance with local guidelines for compensation of research participants, transport costs for all follow-up visits at the hospital will be reimbursed and participants will be given sugar, tea (at each visit) and local currency equivalent of $10 per completed visit (at study completion) ^30, 31^ or following death of a patient.

### Plans for dissemination of outcome and associated data once completed

Feedback to the local academic and clinical community will be given through participation at local research and clinical meetings. A report of the study will be submitted to the local ethical committee who provided ethical permission and communicated to the broader research community via academic presentations (poster and oral) and publications. A follow-up meeting will be convened with the national policy making forum engaged at the start of the study.

Data will be shared within the research community through an open access repository once peer reviewed publication is complete.

### Clinical care

It is anticipated that due to the underlying diagnosis of advanced cancer, the health status of many patients recruited will deteriorate during the study, and several will die as a result of their cancer illness. The role of the study team is not to provide clinical care; however, participants will be advised to use local health services (health centre, district or central facilities) whenever they are found to be unwell and/or with extreme/unrelieved symptoms at the time of a study visit. Responsibility for their care (including any treatment, referrals or admission) will remain with locally available health facilities to preserve the integrity of the study. 

### Distress protocol

A distress protocol will be developed for study staff to alert the principal investigator (or nominated deputy) in the event of extreme distress in participants.

### Recognition and management of risk to study staff

Following initial training, weekly meetings will be held with study staff to check on their work-related wellbeing. The principal investigator will be available by phone to assist study staff whilst they are in the field should they experience any difficulties in the course of their duties. Training delivered by palliative care team members who are experienced in providing bereavement support in local communities will prepare study staff to handle issues around death and dying and how to administer the funeral cost section of the PaCCCt survey. 

### Study limitations

This is a single centre study recruiting patients and their carers from urban and peri-urban settings in Blantyre, Malawi. Patients with four cancer types will be recruited. Study outcomes may have limited generalisability to rural settings and other cancer types. Other common life-limiting illnesses (such as stroke and chronic lung disease) would need separate study due to illness variability in terms of progression, treatment options and outcomes. Generalisability to more well-resourced health settings is also limited, as cancer treatment protocols vary based on availability of resources, e.g. if radiotherapy was available in Malawi, OOPE would potentially increase due to the requirement for multiple hospital visits, though other outcome benefits may also be anticipated.

In common with many studies reporting OOPE ^32^, much of the data relies on accurate self-reporting of information about healthcare utilisation, household income and costs. Patients and carers may for various reasons under or over report these data. Use of trained research field staff and regular meetings with the team during data collection will attempt to optimise the quality of data.

The sample size is likely to be underpowered as a result of using Cohen’s f ^2^. Exposure to palliative care will be based on routine practice and may be insufficient to infer association. It maybe that those choosing not to participate in the study will introduce selection bias in the sample.

### Current study status

Recruitment began in January 2019 and baseline data were collected from 152 households by the end of July 2019. Follow-up is ongoing, due to be completed at the end of January 2020.

## Conclusions

Cancer prevalence and mortality are increasing in many LMICs, including those in the African region. There are currently limited data on healthcare utilisation and related OOPE following a diagnosis of cancer in Malawi, where people are typically diagnosed during an economically productive stage of life with disease already at an advanced stage. During a serious illness and following death, the impact of excessive spending on health continues to be experienced by households, disproportionately so by those already adversely affected by poverty. 

This study will investigate household wellbeing and poverty status in patients receiving a first-time clinical diagnosis for advanced cancer, to explore whether there is evidence that receipt of palliative care can support a reduction in total household expenditure on health whilst maintaining (or improving) wellbeing in households affected by advanced cancer.

## Data availability

### Underlying data

No data are associated with this article

### Extended data

Open Science Framework: Safeguarding the Family. https://doi.org/10.17605/OSF.IO/MDN7K ^22^

- Patient information sheets English Chichewa.docx
- Consent forms patient carer English Chichewa to send.docx
- Patient and carer eligibility criteria.docx
- PaCCCt survey English and Chichewa.docx
- IPOSv1_ChichewaMLW.doc

Data are available under the terms of the Creative Commons Zero “No rights reserved” data waiver (CC0 1.0 Public domain dedication).
