# Supplementary material for: Study protocol for a single-centre observational study of household wellbeing and poverty status following a diagnosis of advanced cancer in Blantyre, Malawi - ‘Safeguarding the Family’ study
Source: Wellcome Open Res. 2020 Mar 2;5:2. Originally published 2020 Jan 7. [Version 2] doi: 10.12688/wellcomeopenres.15633.2 (PMC7047920; doi:10.12688/wellcomeopenres.15633.2)
Supplement: Peer review history

# Peer review: Reviewer response for version 2

**DOI:** 10.21956/wellcomeopenres.17279.r38061

**Published:** 2020-04-01

**Authors:** Charles Normand

**Affiliations:** 1 Centre for Health Policy and Management, Trinity College Dublin, Dublin, Ireland; 2 Cicely Saunders Institute, King's College London, London, UK

This is a useful study and should provide important understanding of the financial impact on families of people with cancer diagnoses in low income countries. The main study tools have been chosen appropriately.

 I remain sceptical about the likely value of the EQ5D3L data in this study. Previous uses in palliative care have tended not to show changes despite other evidence of change in quality of life, and in particular the three level version may not be calibrated in a way that is likely to demonstrate change. There is still no robust evidence on the use of the newer five level version in palliative care, so at this stage it is not useful to recommend a change to the newer version.

 The person with cancer in the study may have a number of different roles. It might be useful to explore the possibility of assessing the different effects on families where the person with cancer is a main bread winner, and where the family impact may be directly affected by income loss as well as the out of pocket expenditure.

I confirm that I have read this submission and believe that I have an appropriate level of expertise to confirm that it is of an acceptable scientific standard.

# Peer review: Reviewer response for version 2

**DOI:** 10.21956/wellcomeopenres.17279.r38062

**Published:** 2020-03-03

**Authors:** Joseph Clark

**Affiliations:** 1 Wolfson Palliative Care Research Centre, Hull York Medical School, University of Hull, Hull, UK

I commend the authors for taking points raised seriously and addressing them accordingly. Clarifications made in v2 of the manuscript address the minor concerns I had with the manuscript which I now approve without reservation.

 I think this is an important area of study for Malawi and elsewhere. It is a study well worth emulating in other countries of the world, to provide longitudinal data, relating to costs incurred relating to cancer over time and any potential impact that access to palliative care has upon this. I look forward to the results of the study.

I confirm that I have read this submission and believe that I have an appropriate level of expertise to confirm that it is of an acceptable scientific standard.

# Peer review: Reviewer response for version 1

**DOI:** 10.21956/wellcomeopenres.17128.r37652

**Published:** 2020-02-26

**Authors:** David C. Currow

**Affiliations:** 1 IMPACCT (Improving Palliative, Aged and Chronic Care through Clinical Research and Translation), Faculty of Health, University of Technology Sydney, Sydney, Australia

The study of out of pocket expenses in low-and-middle income countries is a tremendously important area of research. The investigators are to be commended for their proposal.

 Self-reported out of pocket expenses collected prospectively on the three most prevalent cancers in the community is an good approach to the problem. Complimenting this with an internationally standard measure of quality of life (EQ-5D-3L) is an excellent initiative. The other data collection is feasible and desirable.

 This takes into account the ethics of conducting studies in such populations. The one highly sensitive issue relates to asking a surviving kinsperson to quantify funeral costs.

 It may be of interest to consider including some focus groups for households that have gone from being financially stable to in poverty by the definitions that were used in this study.

I confirm that I have read this submission and believe that I have an appropriate level of expertise to confirm that it is of an acceptable scientific standard.

# Peer review: Reviewer response for version 1

**DOI:** 10.21956/wellcomeopenres.17128.r37624

**Published:** 2020-02-03

**Authors:** Charles Normand

**Affiliations:** 1 Centre for Health Policy and Management, Trinity College Dublin, Dublin, Ireland; 2 Cicely Saunders Institute, King's College London, London, UK

This is an important study and will help to fill the gap in evidence from low income countries. My main concerns are that the 'treatment', i.e. palliative care use is not well specified. High income country evidence suggests timing and form of palliative care is crucial for effects on treatment trajectories and costs as shown in several papers by May *et al*. It also shows that the impact of palliative care interventions are highly sensitive to multimorbidity status.

 Experience suggests that EQ5D3L is unlikely to yield any very interesting data but there is no harm in trying again.

I confirm that I have read this submission and believe that I have an appropriate level of expertise to confirm that it is of an acceptable scientific standard, however I have significant reservations, as outlined above.

# Response

**Published:** 2020-02-26

**Authors:** Maya Bates

I write on behalf of my co-authors to thank the reviewers for their responses to v1 of the above article. Please see our point-by-point response below.

A revised version is being submitted. We look forward to your response. 

**RESPONSE TO REVIEWER 2**

This is an important study and will help to fill the gap in evidence from low income countries. My main concerns are that the 'treatment', i.e. palliative care use is not well specified. High income country evidence suggests timing and form of palliative care is crucial for effects on treatment trajectories and costs as shown in several papers by May *et al*. It also shows that the impact of palliative care interventions are highly sensitive to multimorbidity status.

- 1.Thank you for your comments. After considerable discussion during the planning phase of the study, a descriptive cohort study design was chosen in order to establish a cost and wellbeing dataset reflective of ‘real world’ practice. Future interventional studies will be necessary to see if the findings highlighted from high resource settings are reproduced in low income countries.
  Your concerns about lack of specificity of what constitutes ‘treatment’ are noted. In order to point to additional material relevant to this, we have added the descriptor ‘tertiary level’ to the manuscript:
*pg.4:  Tertiary level palliative care services have been established for adults and children for over fifteen years, and are delivered through in-patient referral, out-patient clinics, and community based care), *
  This descriptor is referenced to open-source standards for palliative care in African settings (African Palliative Care Association APCA Standards for providing quality palliative care across Africa).

Experience suggests that EQ5D3L is unlikely to yield any very interesting data but there is no harm in trying again.

- Few validated wellbeing scores exist for this patient population in Malawi. The EuroQol referenced Chichewa translation of EQ-5D-3L is probably the most widely used score used locally amongst patients with chronic disease conditions (e.g. HIV, TB). Comparison with the Integrated Palliative care Outcome Score (IPOS) as a second patient-reported outcome tool will be of interest.
  In our earlier qualitative work we explored household concepts of wellbeing of relevance to the local population.

# Peer review: Reviewer response for version 1

**DOI:** 10.21956/wellcomeopenres.17128.r37636

**Published:** 2020-01-31

**Authors:** Joseph Clark

**Affiliations:** 1 Wolfson Palliative Care Research Centre, Hull York Medical School, University of Hull, Hull, UK

Thank you for the opportunity to review this paper, which is a protocol for an observational study looking at household expenditure on health, in the context of a cancer diagnosis. The study aims to explore the relationship between receipt of palliative care, total household out-of-pocket expenditure on health and wellbeing following a first-time diagnosis of advanced cancer.

 Overall, the paper is well written and the study addresses a matter of great importance, both for cancer patients in Malawi and elsewhere. I recommend indexing of this manuscript, although ahead of this, there are one or two issues which I feel need to be clarified. I have provided detailed comment on sections of the manuscript below, but my two main areas of concern relate to the following.

 First, given known problems of access to palliative care and the relatively small sample size, I would suggest that the authors need to include comment on the amount of households *likely *to be in receipt of palliative care. What proportion of the included households are likely to receive a palliative care referral? The authors do helpfully outline relevant health infrastructure in setting. I am therefore confused by the note in limitations that “Exposure to palliative care will be based on routine practice and may be insufficient to infer association.” I think this sentence needs clarification. Does this refer to patients who have received a palliative care referral?

 Second, there is some seemingly interchangeable use of terms such as “cancer” and “advanced cancer”. As it becomes clear that the patient population *is* advanced cancer, I think that this warrants justification in the introduction. Was this population chosen as financial expenditure becomes particularly problematic in the context of a diagnosis of advanced cancer (as opposed to cancer)? I think it could also be clearer whether participants to be included received their diagnosis of advanced cancer in the context of a previous diagnosis and advancement of the disease, or would be learning for the first time that they had cancer.

 This is particularly important as we know that worldwide, many cancer patients continue curative therapies long after they may be expected to be effective. Some comment would be helpful regarding potential different financial consequences for families receiving a diagnosis of advanced cancer, compared to those receiving a diagnosis at an earlier stage. Also, if patients are diagnosed *for the first time* with an advanced cancer, how likely is it that families will receive a palliative care referral? What would otherwise trigger a referral? Additionally, following some concerns, I spoke with a clinical doctor colleague regarding the diagnostic criteria of advanced cancer (Table 1). The inclusion of Kaposi’s sarcoma and hepatocellular carcinoma as advanced cancers are clear. However, inclusion of patients with a first time diagnosis (of cervical/oesophageal) with no staging seems vague and could potentially distort the study population. 

 Overall, I think these are issues mostly of clarification and not significant problems with the study or the manuscript. Further comment is provided on individual sections below. If a section is not listed, I have no comment.

**Introduction**

- Unclear to me what ‘default rates’ refer to.
- There is a comma missing following ‘health’ in the Lancet quote (paragraph 2).
- Sentence relevant to SDGs need clarification: “facilitating progress towards the Sustainable Development Goals including – and beyond – health” should this be ‘health improvement?’
- Rationale for the study is clearly justified.

**Protocol**

 The authors may wish to consider additional ways in which participants can provide informed consent (e.g. written **or **observed?), is literacy likely to be an issue? I note that the inclusion criteria is provided as Extended data, but I think key aspects of inclusion should be in the manuscript (e.g. adult population). Including adults only is justified, but different issues present for families where a child receives a cancer diagnosis and in the context of focussing upon ‘households’ should be noted.

**Study tools**

 I commend the authors for work undertaken to date in relation to development of data collection tools. Relating to the PaCCCt, the authors state this will be conducted “from time of onset of symptoms” at baseline. Baseline is not likely to be the time of ‘onset of symptoms’ given the population of the study?

**Secondary outcomes**

 Others will be able to comment more knowledgeably than I on statistical approaches suggested. I would suggest that the authors need to include comment on the amount of households *likely* to be in receipt of palliative care. What proportion of the included households are likely to receive a palliative care referral?

**Loss to follow up**

 Reimbursement of research costs to participants looks appropriate. It may be worth clarifying if families will receive such compensation in the event of patient death (i.e. does ‘death’ count as study completion?).

**Current study status**

 One hundred and fifty two households have already been recruited, which exceeds the stated sample size of 138. Please clarify.

I confirm that I have read this submission and believe that I have an appropriate level of expertise to confirm that it is of an acceptable scientific standard, however I have significant reservations, as outlined above.

# Response

**Published:** 2020-02-26

**Authors:** Maya Bates

I write on behalf of my co-authors to thank the reviewers for their responses to v1 of the above article. Please see our point-by-point response below.

A revised version is being submitted. We look forward to your response. 

**RESPONSE TO REVIEWER 1**

Thank you for the opportunity to review this paper, which is a protocol for an observational study looking at household expenditure on health, in the context of a cancer diagnosis. The study aims to explore the relationship between receipt of palliative care, total household out-of-pocket expenditure on health and wellbeing following a first-time diagnosis of advanced cancer.  Overall, the paper is well written and the study addresses a matter of great importance, both for cancer patients in Malawi and elsewhere. I recommend indexing of this manuscript, although ahead of this, there are one or two issues which I feel need to be clarified. I have provided detailed comment on sections of the manuscript below, but my two main areas of concern relate to the following.

First, given known problems of access to palliative care and the relatively small sample size, I would suggest that the authors need to include comment on the amount of households *likely *to be in receipt of palliative care. What proportion of the included households are likely to receive a palliative care referral? The authors do helpfully outline relevant health infrastructure in setting. I am therefore confused by the note in limitations that “Exposure to palliative care will be based on routine practice and may be insufficient to infer association.” I think this sentence needs clarification. Does this refer to patients who have received a palliative care referral?

- Thank you for your comments which raise an important area for further study. The following text has been added: Pg. 7 *Receipt of palliative care will be presented as both categorical (yes [any] / no [none]) variables, or as interval data (based on the number of contacts with palliative care services from baseline to six months). Proportion of households likely to receive a referral to palliative care services will be dependent on the routine practice of the assessing clinician. *

In this study referral to palliative care reports routine practice at Queen Elizabeth Central Hospital in Blantyre, Malawi. Referrals can be made by clinicians or nurses, maybe written or verbal, but they are not recorded electronically. It is difficult to comment on the amount of households likely to be in receipt of palliative care; we may want to suggest that all patients with advanced cancer are referred to palliative care, but this was not included in the design of the study (and we know that in practice this is not the case). By study completion we will be able to report how many patients have received palliative care, though there is likely to be a discrepancy between how many were referred and how many actually receive care, and we will not know this.

Second, there is some seemingly interchangeable use of terms such as “cancer” and “advanced cancer”. As it becomes clear that the patient population *is* advanced cancer, I think that this warrants justification in the introduction. Was this population chosen as financial expenditure becomes particularly problematic in the context of a diagnosis of advanced cancer (as opposed to cancer)? I think it could also be clearer whether participants to be included received their diagnosis of advanced cancer in the context of a previous diagnosis and advancement of the disease or would be learning for the first time that they had cancer.

- Thank you for highlighting this. Changes have been made as follows:
  Abstract ***Conclusions*** *: This study will provide data on expenditure for healthcare by households affected by advanced cancer in Malawi. We also explore whether receipt of palliative care is associated with a reduction in out-of-pocket expenditure at household level*.
  p.4. *In this study we will explore the association between receipt of palliative care and total household expenditure on health (as a proportion of total household income), and wellbeing for those who were being diagnosed with cancer for the first time, and who were assessed clinically to have  advanced Kaposi’s sarcoma, cervical or oesophageal cancer or hepatocellular carcinoma at Queen Elizabeth Central Hospital in Blantyre, Malawi.*
  We hope this helps with clarity for the reader.

This is particularly important as we know that worldwide, many cancer patients continue curative therapies long after they may be expected to be effective. Some comment would be helpful regarding potential different financial consequences for families receiving a diagnosis of advanced cancer, compared to those receiving a diagnosis at an earlier stage.

- Previous research from Malawi suggests that 60-80% of patients are diagnosed when their disease is already advanced. Treatment options (including radiotherapy) are very limited or absent in this setting. Future studies maybe required to explore the financial (and other) outcomes for families receiving a diagnosis of cancer at an earlier stage. 

 Also, if patients are diagnosed *for the first time* with an advanced cancer, how likely is it that families will receive a palliative care referral? What would otherwise trigger a referral?

- See comments under point 1. This is as yet unknown.

Additionally, following some concerns, I spoke with a clinical doctor colleague regarding the diagnostic criteria of advanced cancer (Table 1). The inclusion of Kaposi’s sarcoma and hepatocellular carcinoma as advanced cancers are clear. However, inclusion of patients with a first time diagnosis (of cervical/oesophageal) with no staging seems vague and could potentially distort the study population.

- Thank you for taking time to enquire about this. Table 1 (pg 5) reporting diagnostic criteria for advanced cancer, has been revised as follows :
  Cervical cancer ALL patients with a first-time diagnosis on clinical examination by specialist doctor
  AND
  with disease at International Federation of Gynaecology and Obstetrics (FIGO) stage 2 and above
  Oesophageal cancer  ALL patients with a first-time diagnosis on endoscopy by specialist doctor
  AND
  assessed as being inoperable,

Overall, I think these are issues mostly of clarification and not significant problems with the study or the manuscript. Further comment is provided on individual sections below. If a section is not listed, I have no comment.

**Introduction**

Unclear to me what ‘default rates’ refer to.

- This has been changed to ‘treatment abandonment’ as follows: Pg. 3 *For the few patients who are able to access potentially curative cancer therapy, treatment abandonment rates are high* *^5^* *^,^* *^6^* *.’ *

There is a comma missing following ‘health’ in the Lancet quote (paragraph 2

- A comma has been inserted: Pg. 3 *The Lancet Commission on Palliative Care and Pain Relief states that ‘access to palliative care and pain relief is a health, equity and human rights imperative which has been largely ignored in the goal to achieve Universal Health Coverage (UHC)*

Sentence relevant to SDGs need clarification: “facilitating progress towards the Sustainable Development Goals including – and beyond – health” should this be ‘health improvement?’

- The phrase ‘health’ changed to ‘ensuring healthy lives’ to make the reference to SDG 3 (ensuring healthy lives and wellbeing for all at all ages) more explicit.
  Pg. 4 *Interventions aimed at reducing the burden of non-communicable diseases can play a key role in global development, facilitating progress towards the Sustainable Development Goals including – and beyond – ‘ensuring healthy lives’.* *^14^* *^–^* *^16^* *.*

Rationale for the study is clearly justified.

**Protocol**

The authors may wish to consider additional ways in which participants can provide informed consent

(e.g. written **or **observed?), is literacy likely to be an issue?

- The consent forms are prepared in English and in local language (Chichewa). As the reviewer has noted, literacy may be an issue. In such cases, study fieldworkers (certified through Good Clinical Practice training) read the details of consent forms to the participants. If they are willing to provide consent, a witnessed thumbprint is used for those unable to write.

I note that the inclusion criteria is provided as Extended data, but I think key aspects of inclusion should be in the manuscript (e.g. adult population). Including adults only is justified, but different issues present for families where a child receives a cancer diagnosis and in the context of focussing upon ‘households’ should be noted.

-  Have included a statement as follows:
  pg.4 *Recruitment for this study will take place amongst adults (anyone 18 years and above) from in-patient wards and out-patient clinics*

**Study tools**

I commend the authors for work undertaken to date in relation to development of data collection tools. Relating to the PaCCCt, the authors state this will be conducted “from time of onset of symptoms” at baseline. Baseline is not likely to be the time of ‘onset of symptoms’ given the population of the study?

-  As the reviewer suggests, baseline is NOT the same as time of onset of symptoms (baseline is at the time of diagnosis), however at baseline, information on costs, health care utilisation and wellbeing were gathered from households *from the time of onset of symptoms. *The text has been changed as follows:
*Pg. 6* *The PaCCCt survey records details of healthcare utilisation firstly from time of onset of symptoms (reported at the time of diagnosis i.e. baseline visit) and subsequently from the last study visit (for follow up visits).*

**Secondary outcomes**

Others will be able to comment more knowledgeably than I on statistical approaches suggested. I would suggest that the authors need to include comment on the amount of households *likely* to be in receipt of palliative care. What proportion of the included households are likely to receive a palliative care referral?

- See point 1 above. The following text has been added:
  Pg. 7 *Receipt of palliative care will be presented as both categorical (yes [any] / no [none]) variables, or as interval data (based on the number of contacts with palliative care services from baseline to six months). Proportion of households likely to receive a referral to palliative care services will be dependent on the routine practice of the assessing clinician. *

**Loss to follow up**

Reimbursement of research costs to participants looks appropriate. It may be worth clarifying if families will receive such compensation in the event of patient death (i.e. does ‘death’ count as study completion?).

- We have included the following statement:  
  p.8 *local currency equivalent of $10 per completed visit (at study completion or following death of a patient)* *^29^* *^,^* *^30^* *.*

**Current study status**

One hundred and fifty two households have already been recruited, which exceeds the stated sample size of 138. Please clarify.

- The sample size based on medium effect size using Cohens f ^2^ was 55 households, however this increased to 138 considering an estimated 20% LTFU and 50% living outside the area. However it is unclear how many patients might die or default over the 6 month period from baseline, therefore we planned to continue to recruit the maximum number of eligible households within our six month recruitment window in the hope of ending up with at least a minimum number complete dataset (baseline to six months) for the 55 households required.
